# Reconceptualizing the Interaction of Behavior and Environment

**DOI:** 10.1007/s40614-024-00417-1

**Published:** 2024-08-22

**Authors:** Jan Philippe de Haan, Carsta Simon

**Affiliations:** 1 Institute of Psychology, Technische Universität Braunschweig, Brunswick, Germany; 2 Department of Psychosocial Health, University of Agder, Grimstad, Norway

**Keywords:** Response strength, Selection, MLBS, Blocking, Strengthening by reinforcement

## Abstract

The concept of response strength and the process of strengthening by reinforcement are controversial in terms of their explanatory power. We clarify potential theoretical misconceptions following from a strength-based account such as essentialist thinking and circular reasoning. These problems also arise in the practice of latent variable modeling in psychometrics. To solve these conceptual problems, we discuss the Multilevel Model of Behavioral Selection (MLBS; Borgstede & Eggert, 2021) as an alternative theoretical framework. We use blocking from Pavlovian conditioning as an example to demonstrate how the MLBS framework prevents misconceptions arising from strength-based accounts and how it provides a more parsimonious and coherent explanation of the phenomenon. We illustrate the need for precisely defined and theoretically meaningful concepts and offer a reinterpretation of “strengthening by reinforcement.” The reconceptualization in terms of the MLBS renders the concept of response strength superfluous. We conclude by highlighting the importance of theoretical reconsideration, putting aside difficulties that arise when attempting to validate strength by empirical means.

The well-established notions of *strengthening by reinforcement* and the accompanying concept of *response strength* are currently the subject of debate (Cowie, 2020; Palmer, 2009, 2021; Simon et al., 2020). On the one hand, several authors have questioned the general theoretical contribution of response strength to understand behavioral processes (Shahan, 2017; Simon et al., 2020). On the other hand, some consider the concept as practicable and argue that with the help of technological advances it will eventually be possible to overcome its merely hypothetical status and provide empirical measures representing response strength (Palmer, 2021). In this article, we (1) highlight theoretical problems following from the notion of response strength; (2) argue that the debate about response strength cannot be resolved through empirical results but instead requires theoretical reconsideration; and (3) provide a theoretical alternative for explaining behavior change without strength.

The controversy surrounding the theoretical concept of strengthening refers primarily to its explanatory value and to its associationist origin. For example, Baum (2002) and Simon et al. (2020) noted that the origin of response strength dates back to early associationist ideas. Thorndike (1927, p. 212) briefly described the Law of Effect as "what comes after a connection acts upon it to alter its strength...". This notion of “strength” was subsequently adopted and expanded through the concept of “reinforcement”. As Skinner (1976, p. 44) articulated:

Through the process of operant conditioning, behavior with this kind of consequence [positively affecting survival value of the organism], becomes more likely to occur. The behavior is said to be *strengthened* by its consequences, and for that reason the consequences themselves are called "reinforcers."

The term “reinforcer” is closely linked to the idea of (associative) strength and the word “strength” shares some semantic similarity with the word “force” (in the physical sense as in rein*forcer*; see Shahan, 2017).

Despite over 80 years of discussion, the theoretical role of “strength” in behavioral psychology remains unresolved (Cowie, 2020). Shahan (2017, p. 108) notes that strength “still serves as the basis of a common implicit theory of reinforcement for behavior analysis.” Cowie (2020), Shahan (2017), and Simon et al. (2020) provided comprehensive reviews of this longstanding debate. Shahan (2017) pointed out that the term “reinforcer” was borrowed from the field of material sciences and is used metaphorically when applied to behavioral science. He also stated that *strengthening by reinforcement* can be descriptive but not explanatory. This aligns with Skinner (1984, p. 518), who emphasized that the basic datum of a science of behavior is the probability of an emitted response and that the notion of strength adds nothing to the understanding of the phenomenon:

It is no accident that rate of responding is successful as a datum because it is particularly appropriate to the fundamental task of a science of behavior. If we are to predict behavior (and possibly to control it), we must deal with *probability of response*. The business of a science of behavior is to evaluate this probability and explore the conditions which determine it. Strength of bond, expectancy, excitatory potential, and so on, carry the notion of probability in an easily imagined form, but the additional properties suggested by these terms have hindered the search for suitable measures. Rate of responding is not a "measure" of probability, but it is the only appropriate datum in a formulation in these terms.

The core of this conception of response probability as the primary dependent variable is a probability function over a set of behavioral alternatives (behaviors that could be emitted at a given time). In this framework, “reinforcement” is understood as the alteration in the probability of a behavior being exhibited. This alteration is represented by a shift in the function mapping of specific behavioral alternatives to their associated probabilities of occurrence. This framework constitutes a descriptive account of behavioral change, because changes in strength are characterized by empirical observations. Thus, the terms “strength” and “probability of occurrence” could be used interchangeably. Although this approach provides an empirical and therefore descriptive interpretation for strength, it does not delve into the underlying *mechanism* causing these alterations in behavioral probability or response rate. The particular explanatory process remains unspecified.

The descriptive interpretation of strength as response probability appears unproblematic, because it makes no explanatory claims. However, several authors have related additional characteristics of responses to the strength concept. Among others, these characteristics include latency, resistance to extinction (or persistence), and force (or intensity; Cowie, 2020; Killeen & Hall, 2001; Palmer, 2009; Simon et al., 2020). Measurable characteristics of behavior such as rate or persistence are seen as methodological approaches to strength (e.g., Killeen & Hall, 2001; Palmer, 2009). As a result, response probability (and rate) is now no longer synonymous with strength, but becomes one of several empirical measures of strength.

Killeen and Hall (2001) used a factor analytic model to relate different empirical measures to strength. Factor analysis is widely used in psychometrics for modeling psychological attributes. This modeling is based on the assumption that the covariance between observable attributes (e.g., item scores from a questionnaire) is not trivial but rather the product of an underlying latent characteristic. In psychometric theory, it is usually assumed that the latent characteristic causes the empirically found covariance structure of observed characteristics. We use the term “latent” to refer to characteristics or processes that are unobserved, i.e., not directly measured. There is no consensus on the criteria that determine whether a trait or process is inherently unobservable or inherently observable. For a broader discussion of the criteria for observability, Okasha (2002) provides an overview. Killeen and Hall (2001) treated strength as a latent characteristic. Regarding the empirical covariance of different dependent variables obtained through experimental manipulation, they conclude that “these variables are clearly measuring the same thing” (i.e., strength; Killeen & Hall, 2001, p. 118).

Despite their widespread use in psychometrics, both latent characteristics and the methods used to model them (i.e., factor analysis) are controversial in terms of their theoretical justification and their actual explanatory power. Borgstede and Eggert (2022) presented two possible perspectives on latent attributes. First, the “pragmatist view” holds that latent attributes can be understood as a more parsimonious representation of empirical observations (e.g., representation of a covariance structure between questionnaire items by a smaller number of latent factors, as is common in latent variable modeling). In this case, these “data models” are pure descriptions of empirical observations. This view relates to the conception of *intervening variables* suggested by MacCorquodale and Meehl (1948), who denote theoretical terms that are completely derived from empirical variables. Second, the “realist view” holds that latent attributes are actual entities. This perspective entails essentialist ideas that are considered unscientific in modern natural sciences (Borgstede & Eggert, 2022; Palmer & Donahoe, 1992). Essentialism is the idea that phenomena of a certain category have an inherent property or *essence* that makes them belong to the given category or class of phenomena (Borgstede & Eggert, 2022; Palmer & Donahoe, 1992). The problem with this perspective lies in the circular definitions that result from it. As Borgstede and Eggert (2022) pointed out, the essence of a category, which should define the inclusion of phenomena in this category, is itself defined by some common characteristic of the phenomena under consideration. Furthermore, they describe a characteristic that serves as essence as “nothing but a placeholder for something unknown to the observer” (Borgstede & Eggert, 2022, p. 125). This view on latent characteristics relates to the notion of *hypothetical construct*, which was also put forward by MacCorquodale and Meehl (1948). This correspondence exists to the extent that hypothetical constructs “are not wholly reducible to empirical terms; they refer to processes or entities that are not directly observed ...” (MacCorquodale & Meehl, 1948, p. 104).

This criticism regarding the explanatory power of latent characteristics applies to response strength, because it is not directly observed. In this context, a descriptive approach to strength as response probability (or rate) such as argued by Skinner (1984) can be seen as pragmatic. The situation becomes more ambiguous with regard to the approach taken by Killeen and Hall (2001): contrary to the standard notion of psychometrics, strength is not assumed to cause variation in different manifest dependent variables. Instead, the experimentally controlled independent variables (e.g., different schedules of reinforcement) from different experiments are seen as causes (controlling variables). When it comes to the covariance between different dependent variables, the classical psychometric notion is partly reintroduced. Positive covariance between dependent variables is then used as an argument that they “are clearly measuring the same thing” (i.e., strength; Killeen & Hall, 2001, p. 118). From this, the conclusion could be drawn that strength is an intermediate characteristic causing not the variation of dependent variables per se but being responsible for their (empirical) covariation. Thus, an interpretative stance is adopted which remains unclear with regard to the pragmatic-realistic distinction. Killeen and Hall (2001, p. 130) argue:

The static properties are indicators, symptoms if you would, of operant strength. . . . Factor analysis is one method of bringing these indicators into coherence—for defining the scales on the separate measures necessary to achieve unanimity. In the end, it is understanding of the thing they indicate, the hypothetical construct of strength, that is our ultimate theoretical goal, as it was Skinner’s.

Assuming the authors adopt a pragmatic point of view, their perspective differs from that proposed by Skinner (1984). Strength is no longer synonymous with probability (or rate) but becomes a summarizing characteristic with regard to all observed dependent variables. Nevertheless, the overall response rate is considered to be the best indicator of strength as “[r]ates can be used with assurance that they are correlated with a fundamental underlying factor, without having to acknowledge that factor” (Killeen & Hall, 2001, p. 129). In their confirmatory factor model, the overall response rate had a factor loading of 1.0 (Killeen & Hall, 2001). This would question the necessity for strength (the latent variable) in the model, as overall response rate would be an error-free indicator. Following this argument, one returns to the purely pragmatic position that strength is another term for probability (or rate). The question of whether to acknowledge or not acknowledge an underlying factor becomes irrelevant.

Another approach that discusses the status and potential usefulness of response strength is presented by Palmer (2009, 2021). Here, strength is introduced as an interpretative tool for situations in which it is not possible to empirically determine behavioral probabilities. These situations occur, for example, when contingencies, and in turn the sample space (entailing possible behavioral alternatives in a given environment), become complex, tend to vary over time, or are unknown (Palmer, 2021). As a specific example, Palmer (2021) referred to the semantic priming procedure. Here, the central dependent variable is latency whereas the use of rate is judged inappropriate based on methodological considerations (trial length and intertrial intervals keep rate constant). In Palmer's (2009, 2021) approach, strength can also be considered a latent (meaning unobserved) variable that is conceptualized as a continuum. Different behavioral alternatives have a certain position on this continuum and therefore vary in strength. The position on this continuum furthermore determines whether a behavioral alternative is emitted or remains hypothetical. Hypothetical behavior refers to behavioral alternatives that could in principle be emitted by the organism given a specific time and environment but that are not emitted (originally, the term “latent behavior” was used for hypothetical behavior but this usage of “latent” is not equivalent to our usage as “not directly observed”). Palmer (2009) described two thresholds located on the strength continuum, one for the emission and one for the observability of behavior. Here, behavior must at least exceed the threshold of emission (i.e., it must take place) to be observable. On a given occasion, different hypothetical behavioral alternatives compete with each other, most of which are mutually exclusive. Contingencies in the environment then determine the position of competing hypothetical behavioral alternatives on the strength continuum. The position on the strength continuum, and therefore the strength assigned to individual behavioral alternatives, determines which behavior is ultimately emitted (passes the threshold of emission). Palmer (2009) illustrated this with a bottleneck mechanism in that only one behavioral alternative (with the highest strength) prevails and passes the bottleneck (i.e., becomes emitted). The approach presented by Palmer also remains unclear with regard to the pragmatic-realistic distinction. In concluding his discussion, Palmer (2021, p. 496) stated:

I agree to this extent: the concept of response strength as a hypothetical construct plays no role in the experimental analysis of behavior, at least at our present level of technology. However, I do not think it can be conceived of as an intervening variable. It would indeed be both convenient and desirable if we could equate response strength with objective measures such as rate or relative frequency per opportunity.

Supposing a practical perspective on strength, Palmer's (2021) viewpoint differs from Skinner's (1984), because strength is not equated with response probability but instead considered a summary term. However, it also differs from Killeen and Hall's (2001) approach (assuming they take a pragmatic perspective), because they explicitly include the summarized characteristics in their proposed model (indicators in the factor analysis model). Palmer (2021) referred less explicitly to the characteristics that may be described by strength. He conceded that strength “is admittedly an imprecise term” (Palmer, 2021, p. 486). It represents, for example, the influence of complex contingencies with numerous simultaneously acting discriminative stimuli as well as physiological processes such as the change in interneuronal conductivity (Palmer, 2021).

The use of “strength” as a summary term leads to a more ambiguous differentiation from realist positions. This ambiguity arises because there is no one-to-one equivalence or straightforward definition of strength (i.e., equating strength with response probability). Furthermore, the absence of an explicit theoretical mechanism facilitates a potential realist fallback on strength or strengthening by reinforcement. An (implicit) realist interpretation of strength could therefore originate from an attempt to overcome this absence.

In the following, a few examples will illustrate how response strength is used in emprirical studies. McLean et al. (2012) examined pigeons under multiple random-interval schedules. In their investigation “response strength was assessed in each condition using both steadystate response rate and resistance to change” (McLean et al., 2012, p. 53). This implies using “strength” as a summary term from a pragmatic perspective, without a clear distinction from a realist perspective. Likewise, Thomason-Sassi et al. (2011) investigated the suitability of latency as a measure of response strength for problem behavior in applied settings. They referred to latency as “one dimension of response strength that is not based on response repetition within a session...” (Thomason-Sassi et al., 2011, p. 52). Again, a pragmatic perspective on “strength” as a summary term may be implied but was not explicitly stated.

McNamara et al. (2015) investigated the acquisition of lever pressing with delayed reinforcement in rats. They also used the terms “strength” and “strengthening by reinforcement” to refer to changes in response rate. Whether a purely pragmatic perspective is adopted, which equates rate and strength as in Skinner (1984), remains unspecified. Ginsburg and Lamb (2013) studied recovery and relapse of ethanol consumption in rats in the context of alternative sources of reinforcement. Here, the term “strength” and the notion of strengthening to describe behavioral frequencies across sessions were used as well. Similar to McNamara et al. (2015), a pragmatist positioning with regard to strength was not explicitly stated.

Owen and Rodriguez (2024) investigated conditions for effectively establishing autoclitics in autistic children’s verbal behavior. They defined strength as follows: “The strength of a [verbal] response, as a metaphor, is an algebraic summation of the effects of concurrent variables, some of which supplement the response, whereas others compete with the response” (Owen & Rodriguez, 2024, p. 205). This approach is similar to Palmer's (2021) perspective, where strength can be understood as an interpretative tool that represents the combined effects of various influences. A pragmatic perspective is solely implied by identifying strength as a metaphor. These examples illustrate both the lack of precision in the use of strength, and the failure to exclude realistic positions. The latter is particularly relevant for the use of strength as a summary term. Thus, implicit realist interpretations of strength will remain possible (cf. Palmer, 2021).

Taking a realist perspective on response strength would entail assigning explanatory power to the construct. On a structural level, a realist interpretation of strength would be conceptualized as a one-dimensional entity whose expression determines the probability of a certain response’s characteristics (probability, frequency, rate, intensity, etc.). Hence, this concept could be easily replaced with any other hypothetical construct (in the sense of MacCorquodale & Meehl, 1948) carrying the idea of a unique dimension such as “motivation” or “association”. From a logical point of view these constructs would fulfill the same purpose in the discussion. It could be argued that the latter concepts do not find great acceptance among radical behaviorist positions, whereas strength does so because it originates from a purely descriptive notion that has been extensively used in the scientific community. Thus, strength as a latent characteristic does not provide an explanatory mechanism for behavior change, regardless of whether a pragmatic or an (implicit) realistic perspective is taken. Assuming that strengthening by reinforcement per se constitutes an explanatory mechanism would be detrimental to a natural science objective, because it does not represent an existing empirical process. Rather, it would lead to the loss of purely descriptive, i.e., pragmatic interpretations of response strength as well as to the "[elevation of response strength] in status from descriptor to explanation" (Simon et al., 2020, p. 683).

The discussed theoretical problems with essentialism, the inconsistent meaning of the term “strength,” and the lack of explanatory power stem primarily from the absence of an explicit theoretical explanation. Therefore, it is indeed desirable to uncover the *mechanism* responsible for altering the aforementioned probability function that determines the occurrence of specific behaviors and to establish a comprehensive theoretical framework for understanding behavior change.

## How to Move Forward

It might be necessary to fundamentally reconsider the theoretical framework for understanding behavioral processes, moving away from the strengthening metaphor rooted in the material sciences (cf. Shahan, 2017). Several authors have advocated for an integration of behavior analysis with biology including the principles of evolutionary theory (Baum, 2002, 2017; Borgstede, 2020; Shahan, 2017; Simon & Hessen, 2019). This connection would provide a common biological foundation for scientific discourse with behavior as its central subject (e.g., Catania, 2013). Muthukrishna and Henrich (2019) explicitly emphasized the importance of evolutionary theory as an overarching theoretical framework for (human) behavior. According to them, a general theory of behavior requires such an overarching theoretical framework (Muthukrishna & Henrich, 2019).

If we view organisms as biological systems and consider behavior as part of an organism’s phenotype, then a connection to evolutionary principles and mechanisms is reasonable. The mechanism of selection is particularly significant. Selectionist conceptions have generated various discussions in the behavioral sciences (e.g., Baum, 2017; Donahoe et al., 1993; Simon & Hessen, 2019) and have been established in radical behaviorist discourse for decades (Skinner, 1981). The principles of natural selection such as variation, inheritance, and differential selection are applied to behavior. Referring to explicit mechanisms such as selection could provide clarification in the ongoing strengthening debate. One particular framework representing a selectionist account is the so-called Multi-Level Model of Behavioral Selection (MLBS, Borgstede & Eggert, 2021), which formalizes selection effects acting on behavioral allocation. We use the term “formal” for expressions or models that are abstracted from natural language. These are in most cases mathematical but could also be logical or computational. By employing formal expressions and avoiding natural language, precision is inherently increased. Therefore, formal models are deemed highly important for developing and integrating theories (Muthukrishna & Henrich, 2019). In the following, we introduce the MLBS along with a demonstration of how different concepts from behavioral psychology can be expressed in terms of its vocabulary. Next, we present how an explicitly defined and biologically informed mechanism offers a reasonable interpretation and prevents misconceptions concerning behavioral phenomena. We illustrate this with a discussion of the blocking effect (Kamin, 1969).

## The Multi-Level Model of Behavioral Selection

The MLBS builds upon the mechanism of selection (Borgstede & Eggert, 2021). It is nevertheless not the first model formalizing behavioral change in such a framework. Both a computational model proposed by McDowell (2004) and a formal mathematical approach stated by Baum (2017), based on the Price Equation (Price, 1970), describe behavioral dynamics from a selectionist perspective. These include, for example, changes in behavioral allocation over time to concurrent variable interval (VI) schedules. They both provide a formal representation of a selection mechanism applied to behavior. The theoretical perspective adopted in these approaches consists primarily of the assumption that behavioral change results from a process that operates in a manner akin to natural selection. In other words, the principles according to which natural selection functions are utilized to explain behavioral change (cf. Borgstede & Eggert, 2021). Therefore, different behavioral alternatives of a single organism are treated *analogously* to individual members of a population in natural selection. From such a perspective, the conditioning of a pigeon in an operant chamber, for example, could be interpreted as follows: The pigeon’s key-pecking is followed by a food pellet, which is assumed to be a “reinforcer". The consequence of the key peck (food pellet) positively selects the behavioral alternative that produced the corresponding consequence over other behavioral alternatives (e.g., wing flapping). Selection hereby refers to the increasing behavioral probability of showing the corresponding behavioral alternative in the future (key-pecking rate increases). By simply transferring the selection mechanism to the behavioral domain, no formal integration is achieved between behavioral selection and natural selection. In contrast, the MLBS does not merely involve the transfer of principles from evolutionary biology to the behavioral domain. Instead, the MLBS is the first formal model explicitly combining behavioral selection and natural selection (Borgstede & Eggert, 2021). This is achieved by relating behavioral variability to expected gains or losses in fitness on the organismic level.

The MLBS also uses the formalism of the Price Equation, which describes selection processes on an abstract mathematical level using the concept of covariance. Covariance represents the statistical relationship of two (random) variables. A positive covariance means that high values of one variable are associated with high values of the other variable. A negative covariance exists when high values of one variable are associated with low values of the other variable. Applied to the example of population genetics, the Price Equation shows that the mean change in an allele frequency between a parent generation and an offspring generation can be decomposed into two additive terms: the covariance between the genotype and fitness of the parent generation and an expectation term of the change from parent to offspring generation (Borgstede & Eggert, 2021; Borgstede & Simon, 2024). The covariance term captures change due to natural selection, whereas the expectation term represents change due to other influences such as mutation (Borgstede & Eggert, 2021). Consider an example based on Simon and Hessen (2019): within a giraffe population with two generations (parent and offspring generation), the length of the neck varies across individuals. If one observes the neck length of all giraffes, one may find that the average neck length in the offspring generation is greater than the average neck length in the parent generation. For the sake of simplicity, it is assumed that a particular allele is associated with a longer neck. The allele associated with the phenotype of a longer neck is now more frequent in the offspring generation than in the parent generation. The Price Equation expresses the mean change in frequency of the specific allele associated with a longer neck between the two generations due to natural selection. The influence of natural selection on the mean change in frequency is then given by the covariance of the considered genotype (associated with longer necks) with fitness in the parent generation (covariance term). In addition, the Price Equation also represents changes not related to natural selection (expectation term). In this example, the covariance between genotype and fitness is positive, i.e., the genotypes under consideration are positively related to fitness. Thus, the change in neck length regarding the offspring generation is positive. This may be because giraffes with long necks are more effective feeders, because they find food in treetops that in turn is associated with higher reproductive success.

By extending the Price Equation to the behavioral level, the MLBS formally states behavioral selection as part of natural selection. According to Borgstede and Eggert (2021), “reinforcement learning can be described as a Darwinian process where the units of selection are individuals showing behavioral variability and the target of selection is the relative allocation of behavior over time within a specified context” (p. 2). The proposition that behaving organisms constitute the unit of selection distinguishes the MLBS from existing selectionist approaches. Traditional selectionist approaches usually focus on the selection of behavior (or behavioral responses) without an explicit connection to fitness on an organismic level (e.g., McDowell, 2004). Borgstede and Eggert (2021) established this connection by explicitly referring to fitness at the organismic level. Furthermore, the MLBS takes a molar perspective on behavior (Borgstede & Eggert, 2021). This perspective means considering behavior as temporally extended on different time scales and across different contexts (see, e.g., Baum, 2002, 2012b; Simon et al., 2020).

A basic principle that can be derived from the MLBS is the so-called *Covariance Based Law of Effect* (CLOE; Borgstede & Eggert, 2021; Borgstede & Luque, 2021). Borgstede (2021, p. 4) explained the CLOE as follows: “The change in behavior due to behavioral selection is proportional to the covariance between behavior and a fitness predictor, and proportional to the statistical effect of the fitness predictor on evolutionary fitness.” In the formal model, the latter effect represents a scaling factor of the covariance term. Hence, the selection effect on behavior is higher the more “fitness-relevant” the fitness predictor is (for the mathematical formulation and derivation of the CLOE, see Borgstede & Eggert, 2021). “Fitness-relevant” in this case means the amount of expected gain (or loss) in fitness the fitness predictor is associated with. In the MLBS framework including the CLOE formalism, fitness is defined as the organism’s survival (Borgstede & Eggert, 2021).

In taking a closer look at the CLOE, the concept of a statistical fitness predictor (SFP or “fitness proxy”) is crucial because it states a theoretical definition of what would traditionally be considered a “reinforcer”. As Borgstede and Eggert (2021, p. 6) noted: “The covariance based law of effect provides us with a general definition of a reinforcer as a context-dependent fitness predictor.” Let us consider an example of a pigeon in an operant chamber. If the pigeon’s pecking on a disc and the subsequent receipt of food pellets covary, this results in selection of pecking, provided the food can be regarded as an SFP (i.e., food uptake covaries with an expected gain in fitness). The selection of pecking results in an increased allocation of time to pecking (i.e., the rate of pecking increases). The same can be applied to the respondent case: consider a rat undergoing a conditioning procedure where a tone precedes an electric shock. The shock can be regarded as an SFP that covaries with the rat’s behavioral response (e.g., freezing) and with the auditory stimulus. The stimulus becomes a predictor for the SFP (i.e., the shock), which is assumed to be negatively related to the organism’s fitness. As a result, freezing is selected over behavioral alternatives in the presence of the auditory stimulus. In both scenarios, the covariance, the statistical relationship with fitness (defining SFPs), and selection play pivotal roles. Moreover, this principle remains valid regardless of whether SFPs predict an expected increase or an expected decrease in fitness.

In general, SFPs incorporate the predictive/signaling properties of reinforcers and make them explicit (e.g., Baum, 2012b; Cowie et al., 2011; Krägeloh et al., 2005). This means SFPs obtain their theoretical meaning by means of their formal definition (Borgstede & Eggert, 2021). As a consequence, terms such as signposts/conditioned reinforcers, Phylogenetically Important Events (PIEs), and induction, which have already been proposed in the signaling discussion, can be effectively integrated into the MLBS framework. This can be achieved by applying the CLOE, which is based on the concept of covariance.

The interpretation of conditioned reinforcers (i.e., stimuli predicting primary reinforcers) in terms of predictors was addressed with the concept of signposts (Cowie, 2020; Shahan, 2010). The signpost account can be referred to as one stimulus (the conditioned reinforcer) mediating the statistical relationship between behavior and SFP (Borgstede & Eggert, 2021). In this context, mediation means that the statistical relationship between SFP and behavior is indirect. The conditioned reinforcer is statistically related to the SFP, meaning it predicts the SFP. Thus, behavior is subject to selection by the conditioned reinforcer (i.e., behavioral allocation changes over time). In an experimental setup with a light as a conditioned reinforcer and food pellets as SFPs (“primary reinforcers”), this would mean that light has an effect on behavioral allocation over time (i.e., response rate changes), given that the light is predictive or statistically related to the SFP. This statistical relationship between SFP and light can be established through empirical pairing (Borgstede & Eggert, 2021).

Furthermore, the emphasis on the concept of prediction has led to information-theoretical approaches to behavioral adaptations in the past (e.g., Ward et al., 2013). Borgstede (2021) shows that information-theoretical approaches for the explanation of behavioral adaptations can be incorporated into a selectionist perspective. According to the MLBS, the maximization of information about the environment is not viewed as “an innate tendency to seek information” (Borgstede, 2021, p. 2). Instead, it is a *consequence* of the implied selection process where organisms behave as though they were “minimizing the average surprise (i.e., information entropy) associated with the environmental feedback to their behavior” (Borgstede, 2021, p. 9).

SFPs are closely connected to the concept of PIEs, proposed by Baum (e.g., 2012b, 2016, 2018). A PIE is an event that has a direct impact on survival and reproductive success (Baum, 2012b). In addition, Baum (2018, p. 313) also names the possibility that “events that are phylogenetically important may be behavioral events.” Every PIE can thus also be interpreted as an SFP, because it statistically predicts fitness. The definition of an SFP does not postulate any requirements apart from covariance with fitness (cf. Borgstede & Eggert, 2021). Hence, the central advantage of the term “SFP” over the term “PIE” is that it obtains its meaning through a precise (meaning formal) definition. In addition, Baum (2023b) refers to the term “SFP” as capturing the theoretical meaning of PIEs.

Baum (2012b, 2018) employed the term “induction” to describe changes in behavioral allocation resulting from environmental events. According to Baum (2012b), these events represent either PIEs or events/stimuli related to PIEs. Behavioral alternatives on which more time is spent as a consequence of induction are referred to as induced behaviors or induced activities (Baum, 2012b). Consider the example of simple food presentation in a pigeon’s enclosure. Food can be seen as a PIE. Making the PIE available to the pigeon entails a change in the allocation of the pigeon’s behavior. Thus, more time is allocated to food-related behavioral alternatives, such as pecking (Baum, 2012b). The food is then said to have induced pecking behavior. The concept of induction presented by Baum (2012b) can also be expressed in terms of the CLOE: Induction is said to describe the *effect* SFPs exert on behavioral allocation, which is the effect due to behavioral selection*.*

In summary, the CLOE provides a general theoretical formulation of behavioral adaptation resulting from a biologically informed selection mechanism. Borgstede and Eggert (2021) showed that the CLOE can explain various behavioral phenomena, including matching, response deprivation setting the occasion for reinforcers, and blocking. They all constitute behavioral adaptations resulting from selection (see Borgstede & Eggert, 2021). Below, we will present the reinterpretation of blocking (Kamin, 1969) in Pavlovian conditioning in terms of the MLBS formulated by Borgstede and Eggert (2021). Although the so-called blocking effect is not an example of operant conditioning, it serves as an instructive illustration for our discussion. It highlights why the nonselectionist perspective invites introducing additional properties to the hypothetical term strength*.* The example also shows how a selectionist account omits the introduction of strength, which prevents subsequent theoretical misconceptions. Finally, this example underscores that respondent and operant conditioning are not fundamentally different mechanisms (see also Simon & Hessen, 2019). Within the framework of the MLBS, both categories of phenomena can be viewed as processes of behavioral adaptation resulting from a selection mechanism. In essence, they require the same theoretical concepts for explanation.

### Blocking: Associations or Predictions?

The blocking effect describes "the deficit in conditioned responding to Stimulus X after AX—> US pairings in which [the stimulus] A alone was previously paired with the US" (Miller et al., 1995, p. 363). US stands for “unconditioned stimulus”. This phenomenon posed a challenge to associationist conceptions because the contiguity between X and the US should theoretically lead to the formation of an association between them. To address the absence of responding to X despite its co-occurrence with the US, Rescorla and Wagner developed one of the most famous learning models in psychology (Miller et al., 1995; Wagner & Rescorla, 1972). The Rescorla-Wagner model has made it possible to include additional assumptions about associative values of responses. One assumption is that the amount of associative strength a given US can acquire is limited. Another assumption is that the smaller the discrepancy between (1) the current associative strength acquired by a given US and (2) the potential associative strength the same US could maximally acquire, the smaller the increase in associative strength becomes. This means that the amount by which associative strength increases across consecutive trials becomes less (Miller et al., 1995). These assumptions aim to explain phenomena like blocking while maintaining an associationist perspective. When first devised, the model remained neutral on cognitive mechanisms related to blocking, such as the formation of expectations (Bolles, 1979). This was achieved by focusing on the concept of associative strength and how it changed dynamically across trials. Nevertheless, interpretations of the model often relate to cognitive mechanisms. As Miller et al., (1995, p. 363) pointed out:

The hallmark of the Rescorla-Wagner model is that, as the discrepancy between the current associative value of the CS and the maximum strength of association that the US can support decreases, less conditioning occurs. Consequently, there is a decrease in the trial-by-trial change in the CS-US association. This aspect of the model has been interpreted by some researchers to imply that the amount of learning that occurs on each trial decreases as the US comes to be fully expected on the basis of the CS (i.e., as the difference between the actual and expected US decreases). . . . It is important to note that such descriptions of the model using the language of "expectancy" and "representation" are interpretations; the model itself does not demand that language.

At its core, the model allows the rate of change of strength to vary dynamically. Here, the focus lies on the concept of association, which connects it conceptually to Thorndike’s Law of Effect (Thorndike, 1927). Given the phenomenon of blocking, the mechanism to account for this dynamic change is frequently considered to be the formation of expectations (cf. Miller et al., 1995). The assumption is that the more “expected” a CS precedes a US, the less the CS-US association is strengthened. In contrast, the more unexpected or surprising that association is to the organism, the greater the increase in associative strength. Thus, the Rescorla-Wagner model offers the possibility to maintain certain conceptions: although blocking is initially difficult to explain, it is possible to remain in associationist notions with the help of further assumptions (i.e., allowing dynamic changes in strength). But how valid is the introduction of colloquial terms such as "surprise value" or mental events such as expectations? These terms are readily invited because the most straightforward nonpragmatic (i.e., realist) interpretation of associative strength in the Rescorla-Wagner model would be to regard associative strength as a hypothetical construct. The same happens when hypothetical cognitive mechanisms are assumed to explain behavior.

An alternative approach to preventing these interpretations is to discard fundamental assumptions (i.e., the associationist perspective) and interpret the phenomenon using another theoretical framework (see, for example, Shahan, 2017). From an MLBS perspective, blocking can be explained without introducing additional assumptions and hypothetical constructs or mechanisms. In a blocking procedure, covariance is first established between a stimulus and an SFP (e.g., food) through pairing (Borgstede & Eggert, 2021). Here, the stimulus becomes a mediator for the statistical relationship between behavior and the SFP. In the second step, another stimulus is added to the first one. The second stimulus always occurs together with the one already applied. Both stimuli (first and second) have maximum covariance, because they always occur together. However, the second stimulus does not explain any *additional* variance in the SFP, i.e., it does not predict the SFP any more than the first stimulus already does. In a formal sense, this corresponds to the problem of multicollinearity known from multiple linear regression models, where predictors are not independent of each other. As a result, there is no selection (i.e., behavioral change) with respect to the second stimulus. This *is* the blocking effect. Although the interpretation of blocking in terms of prediction has already been stated (e.g., Rescorla & Holland, 1982), the MLBS provides a consistent account within a selectionist framework that is explicitly linked to evolutionary theory. Thus, the terms “surprise,” “expectation,” and “associative strength” are no longer needed, but can be replaced by simpler and, above all, theoretically meaningful (i.e., formally defined) equivalents. These equivalents include covariance*,* SFPs, and behavioral selection*.* Terms like “surprise” or “expectation” invoke the idea that the “effectiveness” of certain stimuli depends above all on the amount of prior exposure. That is not required from a selectionist perspective. In this case, the important property is the amount of a stimulus’s independently explained variance in SFPs.

### Implications for the Debate on Response Strength

The blocking example helps to illustrate why a modification of basic assumptions, such as those made in associationism, is necessary and how relying on the explicit mechanism of selection enhances our understanding of the phenomenon: The theoretical explanation of blocking from an MLBS perspective is explicitly embedded in an overarching theoretical framework, i.e., evolutionary biology (cf. Muthukrishna & Henrich, 2019). We believe that the benefit of modifying basic assumptions is equally applicable to the broader debate on response strength. As mentioned before, the CLOE provides a theoretical foundation for reinforcement learning (Borgstede & Eggert, 2021). In contrast to realist or pragmatist strengthening accounts, the overarching theoretical framework is fundamentally different as it relies on the mechanism of selection and connects it explicitly to evolutionary biology.

We can reformulate the process of learning through reinforcement: Instead of assuming that response rate increases because of a reinforcer “strengthening” behavior, one can state that the covariance between behavior and an SFP (e.g., food) results in behavioral selection, which consequently results in an increased (relative) allocation of time to that behavior (i.e., the rate of responding increases) on subsequent occasions. The model makes concepts like “strength,” “(strengthening by) reinforcement,” or “reinforcer” superfluous. Borgstede and Eggert’s (2021) analysis is in line with Baum’s (2023a) recommendation that the use of these terms should be discontinued in explanations of behavior. Former questions derived from a strengthening perspective, such as how to map response strength via behavioral indicators, are circumvented (cf. Killeen & Hall, 2001). The model would alter fundamental assumptions and promote a theoretically defined and meaningful vocabulary (cf. Baum, 2023b).

## Difficulties Concerning Empirical Evidence against Strength

A theoretical revision to the concept of strength and accompanying processes, such as the MLBS approach presented here, attempts to change the debate on a theoretical level. In addition to theoretical arguments, empirical evidence has been put forth challenging the necessity of the concept of strength and the associated mechanism of reinforcement. Here, the strengthening perspective is often questioned via the function of reinforcers. From a strengthening perspective, reinforcers strengthen behavioral alternatives (strengthening by reinforcement). Therefore, assuming that reinforcers *signal* upcoming events rather than strengthen certain behavioral alternatives opposes the strengthening perspective.

For example, results from a pigeon experiment by Krägeloh et al. (2005) suggested that reinforcers can be interpreted as signals for the location of subsequent reinforcers. In their study, reinforcement was made contingent upon changing locations after a response was reinforced in one place. The accompanying increased change in the locations of pigeons suggests that their response in one location was not reinforced (i.e., strengthened), because the response in that location did not increase. Instead, the reinforcer was interpreted as signaling the availability of further reinforcers in another location. Cowie et al. (2021) conducted a study similar to Krägeloh et al. (2005) with children and reported similar results. Another study in pigeons by Davison and Baum (2006) further suggested signaling rather than strengthening properties of conditioned reinforcers.

Baum (2012a) also pointed out that a strengthening perspective on extinction is not compatible with the partial-reinforcement extinction effect (PREE). If extinction entails a decrease in the strength of a behavior, then one would expect the behavior to extinguish more rapidly when established through partial reinforcement compared to continuous reinforcement. This is because less strength is assumed to be present under partial reinforcement, because strength is presumed to be proportional to the amount of reinforcement received. However, this is not the case with extinction. Instead, Baum (2012a) proposed that extinction amounts to discrimination, which is consistent with a signaling perspective on reinforcers (or in this case their absence). From a molar perspective, reinforcer rate can be understood as a discriminative stimulus (Baum, 2012a). According to this assertion, extinction speed is inversely proportional to the difference between reinforcer rate before and after extinction began. The greater the discrepancy between the reinforcer rate before and during extinction, the more effectively the organism discriminates. Following this, the organism reallocates behavior previously reinforced, for example, key-pecking, to other behavioral alternatives that have not typically been recorded before (Baum, 2012a). The effectiveness of discrimination determines the subsequent speed of reallocation. As a result, the time to extinction is shorter for continuous reinforcement than for partial reinforcement which explains the PREE.

Simon and Baum (2017) investigated human behavioral dynamics in conversations. Their results on participants’ allocation of speech and gaze do not support strengthening by reinforcement. Their study was a systematic replication of Conger and Killeen (1974) in which participants spoke to two confederates, whose approval of what the participants said, followed different variable interval schedules. Conger and Killeen concluded that the confederates’ agreement reinforced the participants’ allocation of speech to the confederates. However, traditionally, a reinforcer is said to strengthen a response or stimulus–response connection when the reinforcer occurs *contiguously* with the response (Skinner, 1948; Thorndike, 1911/2000). Conger and Killeen did not present the event assumed to strengthen the verbal response (confederates’ approval) in close temporal proximity to the verbal response serving as the dependent variable (participant’s speech). Although they measured time spent looking at a confederate (gaze) when talking as their dependent variable, approval (the putative reinforcer) was delivered for any verbal behavior—that is, independently of gaze. This lapse motivated Simon and Baum to investigate the possibility that Conger and Killeen’s results do not result from strengthening by reinforcement. Simon and Baum’s confederates delivered half of the approval contiguously to participants’ gaze and half independent of gaze. Whether or not the putative reinforcer (confederates’ approval) was delivered contiguously with the behavior assumed to be strengthened (participants’ speech) did not influence participants’ allocation of speech. This result suggests that approval signaled the participants who to talk to, rather than strengthened their allocation of speech.

Wood and Simon (2023)^1^ and Simon and Wood (in preparation) examined transitions of typically developing children and of children diagnosed with autism spectrum disorder to see whether their transitions can most straightforwardly be explained by strengthening of past behavior by reinforcement or by signaling effects of events serving as discriminative stimuli for future behavior. In their studies, children transitioned between playmats, which provided putative reinforcers of varying richness. In one of two conditions, the mats’ colors predicted reinforcer richness. For example, children who preferred to watch videos could always watch a shorter video after going to a green mat, a medium-length video after going to a red mat, and a longer video after going to the yellow mat. In the other condition, mat color was not associated with a certain reinforcer richness. That is, video length was unrelated to mat color. Independent of diagnosis, all children transitioned more quickly to predictable longer videos and more slowly to predictable shorter videos. These differences in transition times were absent when mat color did not predict video length.

Whether the last transition was to a longer or shorter video and the upcoming video length jointly explained differences in transition times, showing divided control by the signal of the upcoming reinforcer context and the previously experienced reinforcer context. If the most recent response had been “strengthened” and one assumes that walking faster reflects more strength of walking behavior than walking slowly, children had walked faster after having seen a longer video and slower after having seen a shorter video. The opposite was observed—transitions after the long videos were longer than after the short videos. This finding is consistent with the Negative Incentive Contrast Effect,^2^ which designates the pattern that performance generally reduces when reinforcer density downshifts (see, e.g., Tinklepaugh, 1928, for an early study in which monkeys happily ate lettuce—a less dense reinforcer, unless they were first given bananas—a denser reinforcer). Both the Incentive Contrast Effect in general and the joint control of upcoming and previous video length in Wood and Simon (2023) and Simon and Wood (in preparation) support that organisms discriminate between relations between events rather than having their behavior strengthened in the sense of being “pushed into the future” by reinforcement.

Although these findings pose challenges to the strengthening framework, Shahan (2017, p. 119) describes response strength as a "potentially unfalsifiable assumption based on an inferred hypothetical process." We agree with Shahan’s (2017) position and advocate for prioritizing overall theoretical consistency when comparing explanatory approaches for behavioral dynamics. When we have different explanations of observed events, the simplest one is usually preferable (principle of parsimony, also known as Occam’s razor).

To highlight the benefits of an integrated evolutionary account, a study in the domain of verbal behavior proposed by Simon (2020) is used below as an example. We demonstrate that potential results assumed from a signaling perspective can be interpreted within a strengthening framework by incorporating additional assumptions. The design was outlined as follows (Simon, 2020, p. 376):

The . . . study makes PIEs available contingent on a participant’s choice of a different alternative than the one previously chosen. A participant and ostensibly two confederates solve short tasks between which they can socialize for a certain time. Confederates provide more PIEs such as smiles, agreement, praise, and nodding, contingent on their not being the confederate with whom the participant talked after the last task, that is, last time they could choose their conversation partner. If participants most often choose to talk to the confederate to whom they talked previously, this suggests that the most recent behavior was strengthened, but if they most often talk to the confederate to whom they did not previously talk to, this suggests that their behavior is under control of the past providing PIEs contingent on switching.

**Fig. 1:**
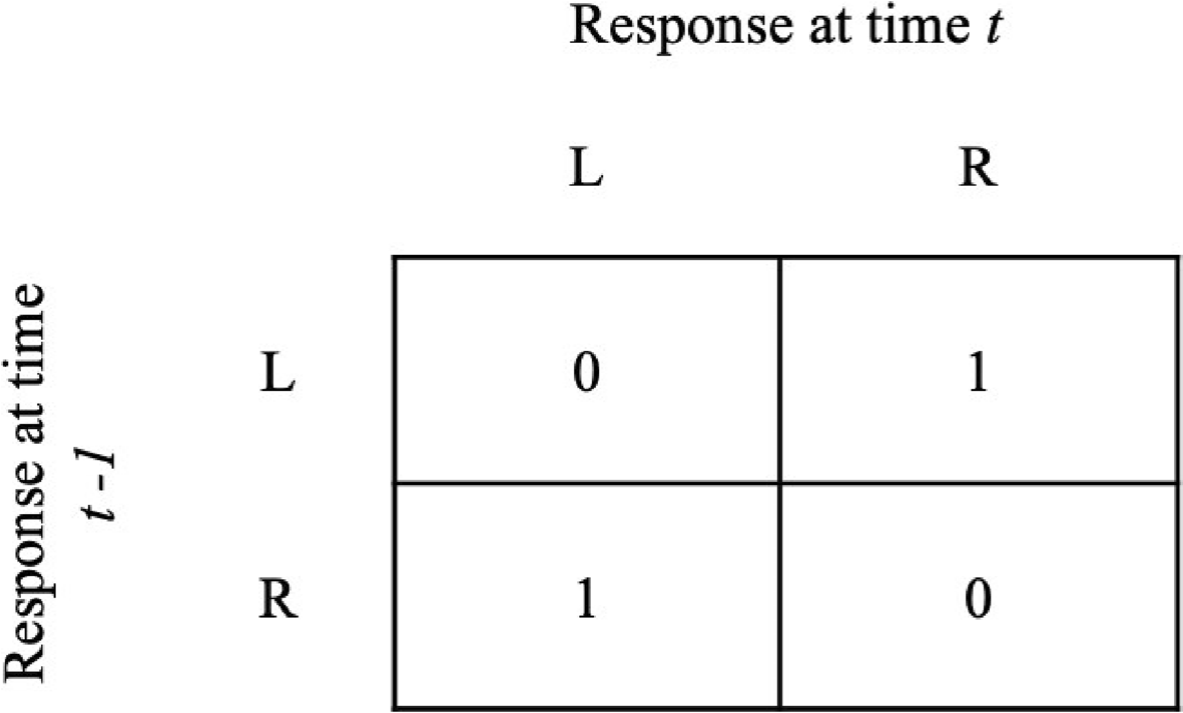
Payoff Matrix for the Contingency Described in Simon (2020)

The contingency described in this study can be abstractly described with a payoff matrix (see Fig. 1). This matrix shows the consequence of choosing either alternative (in this case conversational partners on the left, L or on the right, R) at a time *t* dependent on what was chosen the trial before (time *t − *1). However, this does not apply to the first trial, because the time *t* − 1 would have no meaning in that case.

Number "1" represents verbal “reinforcement” that is received from the conversational partner (a putative SFP). Hence, "0" states that no “reinforcement” takes place in a given trial. From an MLBS perspective, switching is statistically related to (i.e., covarying with) certain verbal SFPs such as smiling or agreeing and therefore selected (i.e., more “switching” occurs). Furthermore, it is possible to go into more detail: “receiving” SFPs from one conversational partner is also covarying with subsequent available SFPs from the other partner. Thus, SFPs from one partner predict SFPs from the other. The phenomenon of switching can be satisfactorily explained in a theoretically consistent manner with MLBS terminology. It is worth pointing out that these empirical observations alone do not contradict the strength perspective, which can be modified to accommodate reinforcement of molar response sequences (see Cowie et al., 2021, for a critical discussion) or treating switching as an operant in its own right (Skinner, 1984). The main benefit of the MLBS account is that it provides an explanation explicitly linked to evolutionary theory. This supports the proposition that “[behavior] analysis is properly [a] part of biology” (Baum, 2018, p. 302).

## Conclusion

A natural science should address the question of a mechanism of behavioral change. Although the usefulness of the response strength concept in the interpretation of behavioral processes is affirmed from a pragmatic perspective (e.g., Palmer, 2009, 2021), it does not provide an explicit explanation and leaves open the possibility for theoretical misconceptions analogous to the role of “surprise” in explanations of the blocking effect. The debate about strengthening may be informed by theoretical reconsiderations. In this context, scientific principles such as parsimony, the formal definition of theoretical terms, and integration into an overarching framework such as evolutionary biology should play a decisive role. Adopting the MLBS explicitly incorporates behavioral selection as a mechanism and offers the potential to theoretically ground the empirical description of behavioral dynamics. The accompanying adjustment of theoretical vocabulary leads to a situation in which the concept of response strength is no longer necessary.

## Data Availability

Data sharing not applicable to this article as no datasets were generated or analyzed.

## Footnotes

1 Note that the interpreation of Wood and Simon’s (2023) and Simon and Wood’s (in press) data given here is slightly different and only partially compatible with the interpretation given in these two publications. This revised interpretation of the studies’ published data was originally suggested by Christopher Podlesnik when serving as an opponent in Wood’s PhD-defense (Wood, 2024).

2 The present discussion of results diverges partly from Wood and Simon (2023). It was first put forward by Christopher Podlesnik at Aleksandra Wood’s (2024) defense of her PhD-thesis.

## Declarations

### Use of Large Language Models (LLMs)

ChatGPT 4/4o and 3.5 were used for language editing to ensure better readability and comprehensibility.
